# University of London

**Published:** 1852-11

**Authors:** 


### UNIVERSITY OF LONDON.

*Somerset House.*

*Examiners.*

*Faculty of Medicine.*—Intellectual Philosophy, Logic, and Moral Philosophy: Rev. Henry Alford, B. D., T. B. Burcham, Esq., M. A.

Medicine: Archibald Billing, M. A., M. D., F. R. S., Alexander Tweedie, M. D., F. R. S.

Surgery: Sir Stephen L. Hammick, Bart., Joseph Hodgson, Esq., F. R. S.

Anatomy and Physiology: Francis Kiernan, Esq., F. R. S., William Sharpey, M. D., F. R. S.

Physiology and Comparative Anatomy: W. B. Carpenter, M. D., F. R. S.

Midwifery: Edward Rigby, M. D.

Chemistry: W. Thomas Brande, Esq., F. R. S.

Botany: Rev. J. S. Henslow, M. A.

Materia Medica and Pharmacy: Jonathan Pereira, M. D., F. R. S.

*Examinations for the Degree of Bachelor of Medicine.*

Candidates for the degree of Bachelor of Medicine are required—1. To have been engaged during four years in their professional studies at one or more of the institutions or schools recognized by this university. 2. To have spent one year at least of the four in one or more of the recognized institutions or schools in the United Kingdom. 3. To pass two examinations.

*First Examination.*

The First Examination commences on the first Monday in August.

The candidate is required to produce certificates—1. Of having completed his nineteenth year. 2. Of having taken a degree in Arts in this university, or in a university the degrees granted by which are recognised by the senate; or of having passed the matriculation examination. 3. Of having been a student during two years at a medical school recognised by this university, subsequently to having taken a degree in Arts, or passed the matriculation examination. 4. Of having attended a course of lectures on each of four of the subjects in the following list: Descriptive and Surgical Anatomy, General Anatomy and Physiology, Comparative Anatomy, Pathological Anatomy, Chemistry, Botany, Materia Medica and Pharmacy, General Pathology, General Therapeutics, Forensic Medicine, Hygiene, Midwifery and Diseases peculiar to Women and Infants, Surgery, Medicine. 5. Of having dissected during nine months. 6. Of having attended a comprehensive course of Practical Chemistry. 7. Of having attended to Practical Pharmacy sufficiently to acquire a practical knowledge in the preparation of medicines. The fee for examination is five pounds. Candidates are examined in Anatomy, Physiology, Chemistry, Botany, (a syllabus of which may be had by application at the office of the university,) and Materia Medica and Pharmacy; they are also required to translate passages from the Latin Pharmacopœia. In the week next following the examination, the examiners arrange in two divisions such of the candidates as shall have passed; and a certificate, signed by the registrar, is delivered to each candidate.

*Examination for Honors.*

Any candidate who has been placed in the first division may be examined for honors, in the week following, in any or all of the following subjects:—Anatomy and Physiology, Chemistry, Materia Medica and Pharmaceutical Chemistry, and Structural and Physiological Botany. If in the opinion of the examiners sufficient merit be evinced, the candidates who distinguish themselves the most in Anatomy and Physiology, in Chemistry, and in Materia Medica and Pharmaceutical Chemistry, will each receive an exhibition of thirty pounds a year for the next two years; and the first and second candidates in each of the preceding subjects, and the first in Structural and Physiological Botany, will recieve a gold medal of the value of five pounds. Certificates of honor are given in each subject.

*Second Examination.*

The Second Examination commences on the first Monday in November. No candidate is admitted to this examination within two academical years of the first examination. Certificates required—1. Of having passed the first examination. 2. Of having subsequently attended a course of lectures on each of two of the subjects in the foregoing list, and for which the candidates did not present certificates at the first examination. 3. Of having, since the first examination, dissected during six months. 4. Of having conducted at least six labors. 5. Of having attended the surgical practice of a recognised hospital or hospitals during twelve months, and lectures on clinical surgery. 6. Of having attended the medical practice of a recognised hospital or hospitals during other twelve months, and lectures on clinical medicine. 7. Of having subsequently attended to practical medicine in a recognised hospital, infirmary, or dispensary, during six months. 8. Of moral character from a teacher in the last school or institution at which he studied. The fee for examination is five pounds. Candidates are examined in Physiology (including Comparative Anatomy); General Pathology, General Therapeutics, and Hygiene; Surgery; Medicine; Midwifery; Forensic Medicine; they are also required to translate passages of the Latin Pharmacopœia into English, and of the English Pharmacopœia into Latin, and to report on the cases of actual patients. In the following week the examiners arrange in two divisions such of the candidates as shall have passed; and a certificate under the seal of the University, and signed by the Chancellor, is delivered to each candidate.

*Examination for Honors.*

Any candidate who has been placed in the first division may be examined for honors, in the week following, in any or all of the following subjects:—Physiology and Comparative Anatomy, Surgery, Medicine, Midwifery. If in the opinion of the examiners sufficient merit be evinced, the candidates who distinguish themselves the most in Physiology and Comparative Anatomy, in Surgery, and in Medicine, will each receive an exhibition of fifty pounds per annum for the next two years, with the style of University Medical Scholar. The first and second candidates in each of the first three of the preceding subjects, and the candidate who distinguishes himself the most in Midwifery, each receive a gold medal of the value of five pounds. Certificates of honor are given in each subject.

*Examination for the Degree of Doctor of Medicine.*

The examination commences on the fourth Monday in November. Certificates required—1. Of having taken the degree of bachelor of medicine in this university, or a degree in medicine or in surgery at a university the degrees granted by which are recognised by the senate. (Those candidates who have not taken the degree in this university must produce a certificate of having completed their twenty-third year.) 2. Of having attended, subsequently to having taken one of the above degrees, (*a*) to clinical or practical medicine during two years in a medical institution recognised by this university; (*b*) or, to clinical or practical medicine during one year, and of having been engaged during three years in the practice of his profession; (*c*) or, if he have taken the degree of bachelor of medicine in this university, of having been engaged during five years in the practice of his profession. (One year of attendance on clinical or practical medicine, or two years of practice, will be dispensed with in the case of those candidates who at the second examination were placed in the first division ) 3. Of moral character, signed by two persons of respectability. The fee for examination is ten pounds.

*Candidates are examined in the following subjects:*—Elements of Intellectual Philosophy, Logic, and Moral Philosophy; Medicine. A Commentary on a Case in Medicine, Surgery, or Midwifery, at the option of the candidate. *Viva voce* interrogations on the answers to the printed papers, and on the commentary. The candidate is also required to report on cases of actual patients. In the following week the examiners arrange in two divisions, each in alphabetical order, such of the candidates as shall have passed; and a certificate, under the seal of the university, and signed by the Chancellor, is delivered to each candidate.

*Regulations relating to Students who commenced their Medical Studies in or before January,* 1839.

*Degree of Bachelor of Medicine.*—Candidates who commenced their professional studies in or before January, 1839, being then not less than fourteen years of age, will be admitted to the first examination for the degree of bachelor of medicine, on producing certificates—1. Of having been engaged during two years in their professional studies. 2. Of having attended a course of lectures on each of four of the subjects comprehended in the foregoing list. 3. Of having dissected during nine months. 4. Of having attended to practical pharmacy.

They will be admitted to the second examination on producing certificates—1. Of having been engaged during four years in their professional studies. 2. Of having passed the first examination. 3. Of having attended a course of lectures on each of two of the subjects in the foregoing list, in addition to those for which certificates were produced at the first examination. 4. Of having dissected during twelve months. 5. Of having attended to practical pharmacy.. 6. Of having conducted at least six labors. 7. Of having attended the surgical practice of a recognised hospital or hospitals during twelve months. 8. Of having attended the medical practice of a recognised hospital or hospitals during other twelve months. 9. Of having completed their twenty-second year. 10. Of moral character, from a teacher in the last school or institution at which they studied. Candidates who have not taken a degree in Arts, or passed the matriculation examination, translate a portion of Celsus *de Re Medica.*

*Regulations relating to Practitioners in Medicine or Surgery desirous of obtaining Degrees in Medicine.*

*Degree of Bachelor of Medicine.*—Candidates admitted to the two examinations on producing certificates—1. Of having been admitted, prior to 1840, members of one of the legally constituted bodies in the United Kingdom for licensing practitioners in medicine or surgery; or of having served, previous to 1840, as surgeons or assistant-surgeons in her Majesty's Army, Ordnance, or Navy, or in the service of the East India Company. 2. Of having received a part of their education at a recognised institution or school, as required by the charter of the university. 3. Of moral character, signed by two persons of respectability. Candidates who have not taken a degree in Arts, or passed the matriculation examination, translate a portion of Celsus *de Re Medica.*

*Degree of Doctor of Medicine.*—Candidates are admitted to examination on producing certificates—1. Of having been engaged during five years in the practice of their profession. 2. Of having taken the degree of bachelor of medicine. Candidates who have not taken a degree in Arts, or passed the matriculation examination, translate a portion of Celsus *de Re Medica.*

In the case of a candidate who fails to pass any one of the examinations, the fee is *not* returned to him, but he may present himself at a subsequent examination without any additional fee.

